# 607. Candidemia: Role of T2Candida® compared to Bact/Alert Virtuo blood culture system in a real-world setting

**DOI:** 10.1093/ofid/ofad500.673

**Published:** 2023-11-27

**Authors:** Navina K Birk, Sana Soman, Nandita Kapur, Vedanth Pochhareddy, William P Dillon, Michael Veve, Linoj Samuel, Mayur Ramesh, George J Alangaden

**Affiliations:** Henry Ford Hospital, Detroit, Michigan; Henry Ford Hospital, Detroit, Michigan; Henry Ford Health System, Detroit, Michigan; Henry Ford Health System, Detroit, Michigan; Henry Ford Hospital, Detroit, Michigan; Henry Ford Health, Detroit, Michigan; Henry Ford Hospital, Detroit, Michigan; Henry Ford Hospital, Detroit, Michigan; Henry Ford Health, Detroit, Michigan

## Abstract

**Background:**

Candidemia is the most common cause of invasive fungal infections with mortality rates up to 60%. The current standard for diagnosis of candidemia is traditional blood cultures (BC) but it has low sensitivity. The need for rapid identification of candidemia has led to the development of non-culture-based diagnostic platforms. T2Candida® (T2) is an FDA approved direct from blood PCR test. T2 detects 5 candida species (*C. albicans/C. tropicalis*, *C. parapsilosis,* & *C. krusei/C. glabrata*) with a turnaround time of three to five hours. T2 is used at our institution for the diagnosis of candidemia in the intensive care units (ICU) if prior blood cultures are negative. Patients with positive T2 results are managed the same as patients with positive BC. In February 2019, our health system switched from the VersaTREK™ to a more sensitive Bact/Alert Virtuo BC system. Our objective was to assess the impact of the new Virtuo system on the diagnosis of candidemia compared to T2 in a real-world setting.

**Figure:**
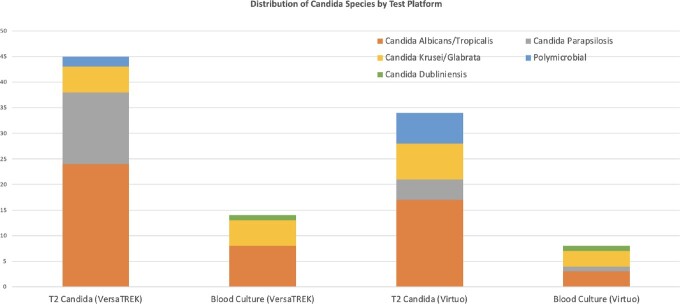

**Methods:**

All T2 and concurrent BC results were retrospectively collected from January 2018 to January 2019 (VersaTREK™ cohort) and March 2019 to March 2020 (Virtuo cohort) in our quaternary care facility in metro Detroit. Only patients with presumed candidemia were included (ICU patients with sepsis, recent exposure to anti-bacterial agents, and negative BC for candida in the past 7 days). Demographic data and the results of T2 and concurrent BC (obtained within 48 hours of T2) were analyzed for the presence or absence of candida. Indeterminate T2 results were excluded. Descriptive statistics were utilized to report the results.

**Results:**

A total of 522 and 348 T2 tests performed with concurrent BC through VersaTREK™ and Virtuo systems respectively were included for analysis. In this ICU cohort with presumed candidemia, T2 remained superior: T2 positivity 45 (8.6%) vs. VersaTREK™ BC positivity 14 (2.7%) (p < 0.001) and T2 positivity 34 (9.8%) vs. Virtuo BC positivity 8 (2.3%) (p < 0.001) (Figure 1). The Virtuo cohort had overall fewer T2 tests performed. This may be because the more sensitive Virtuo system could have detected more cases of candidemia than VersaTREK™ obviating the need for T2 test.

**Conclusion:**

T2 may still have a role in the early diagnosis of candidemia despite the use of newer sensitive blood culture systems.

**Disclosures:**

**Michael Veve, PharmD, MPH**, National Institutes of Health: Grant/Research Support|Paratek Pharmaceuticals: Grant/Research Support

